# Supplementary figures and images for: Host-symbiont stress response to lack-of-sulfide in the giant ciliate mutualism
Source: PLoS One. 2022 Feb 25;17(2):e0254910. doi: 10.1371/journal.pone.0254910 (PMC8880863; doi:10.1371/journal.pone.0254910)

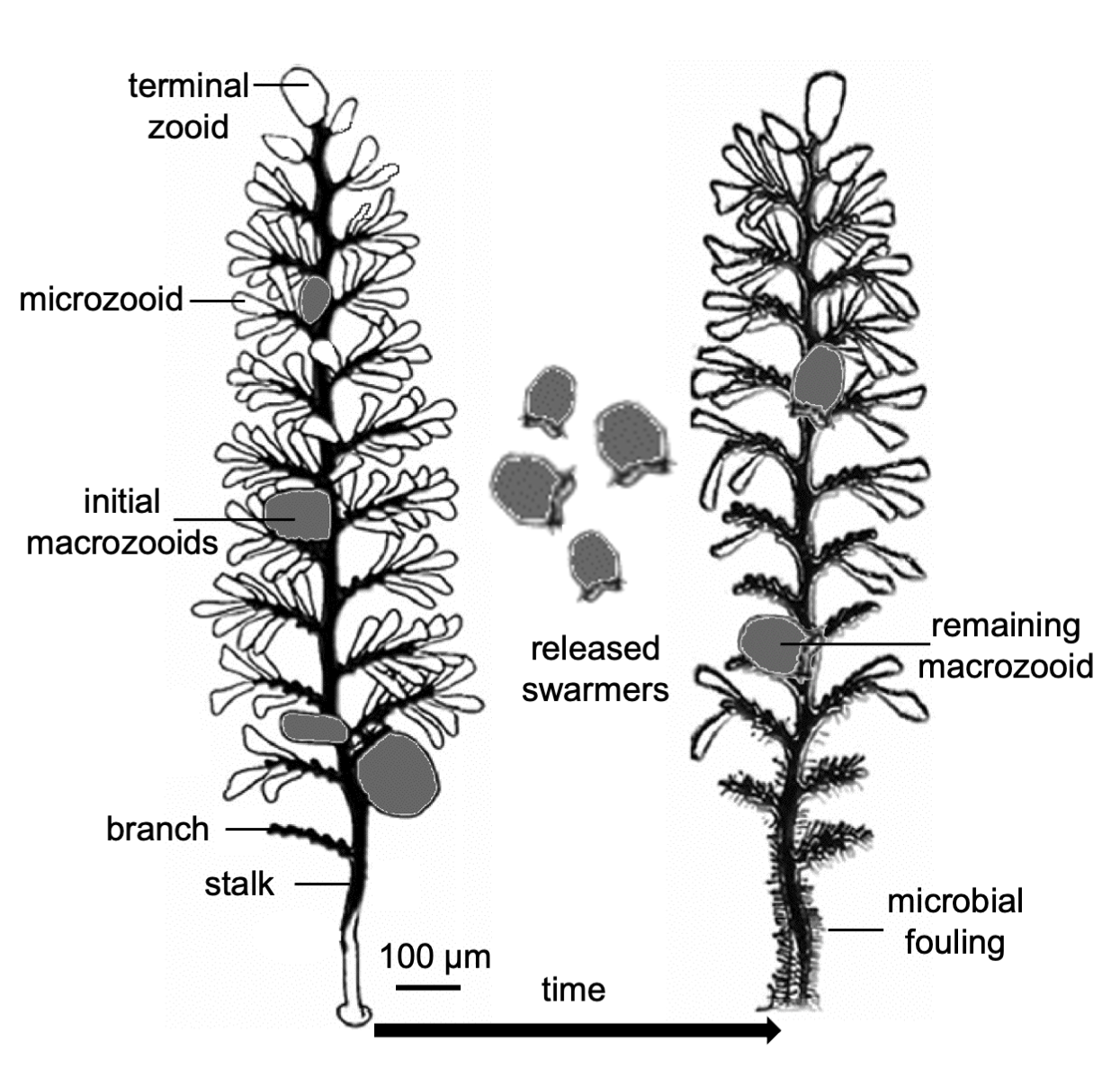

Supplement: S1 Fig — The colony is composed of a stalk with alternate branches and three different cell types–terminal zooids for division, microzooids for nutrition, and macrozooids for asexual reproduction. The size of the colony is counted in number of branches. A colony with initial macrozooids present at the start of the experiment and remaining macrozooids at the end of experiment is shown. During experimental time the release of swarmers was also counted. (TIF) [file pone.0254910.s001.tif]

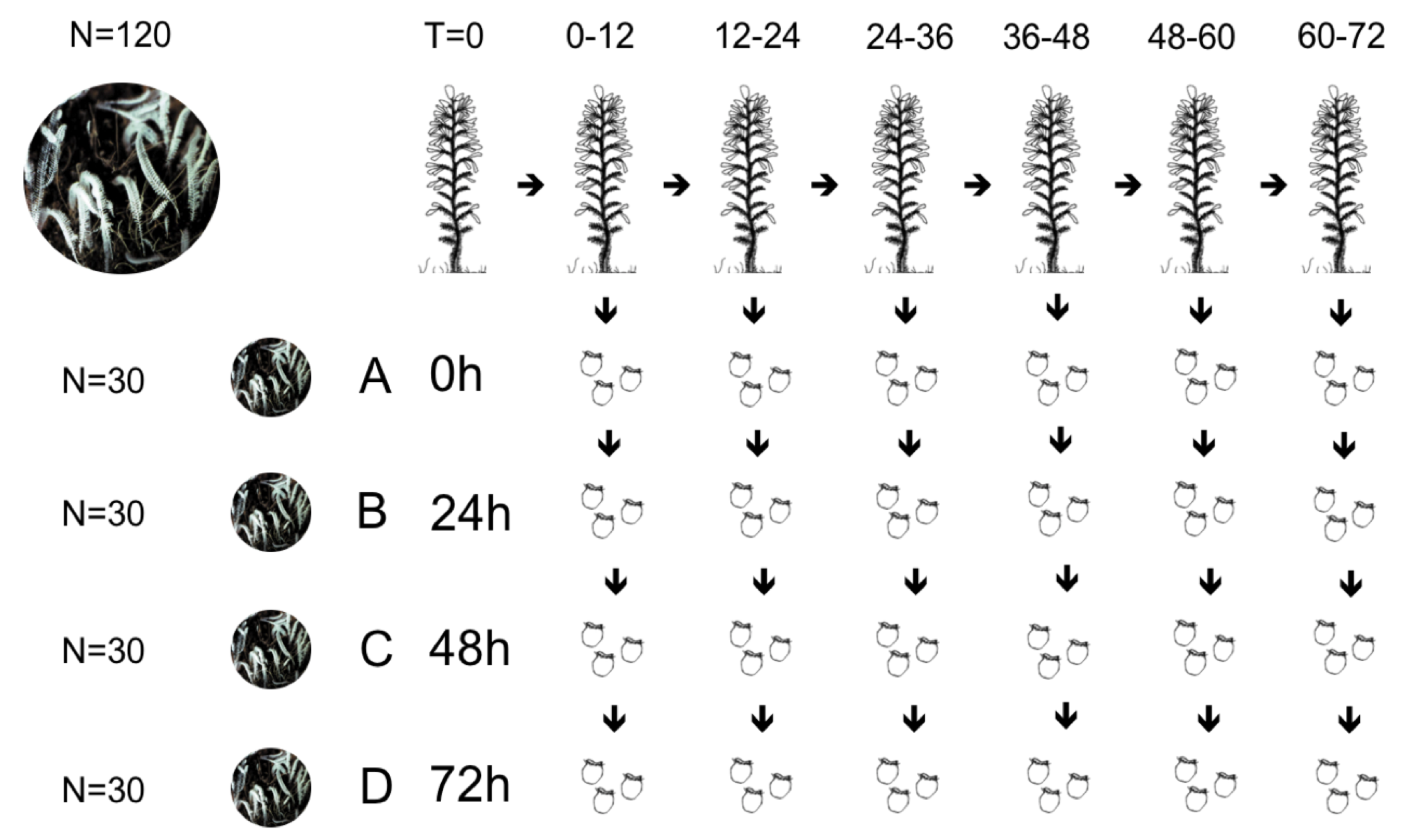

Supplement: S2 Fig — Colonies (n = 120) were monitored every 12 h (horizontal time line). Released swarmers from each of this time points were divided in 4 cohorts (A, B, C, D; vertical time line). (TIF) [file pone.0254910.s002.tif]

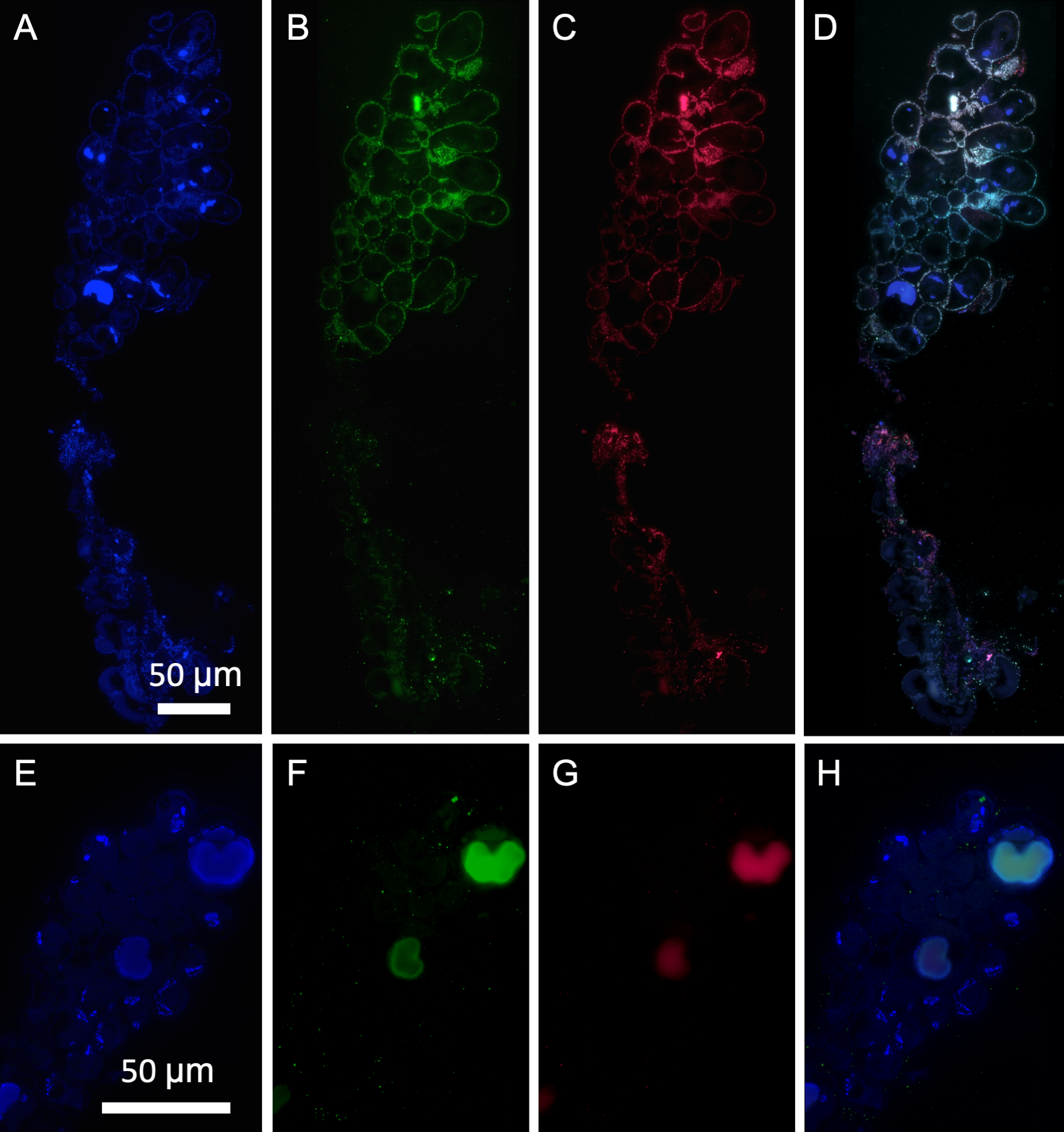

Supplement: S4 Fig — Colony alive after 48 h (A) DAPI staining (blue), (B) symbiont-specific probe (green), (C) EUBmix and Archea probes (red) (D) composite of A, B, C; note the increase in microbial fouling from top to bottom. Colony alive after 72 h with very few symbionts left (E) DAPI staining (blue), (F) symbiont-specific probe (green), (G) EUBmix and Archea probes (red), (H) composite of E, F and G. (TIF) [file pone.0254910.s004.tif]
