# Supplementary material for: Host-symbiont stress response to lack-of-sulfide in the giant ciliate mutualism
Source: PLoS One. 2022 Feb 25;17(2):e0254910. doi: 10.1371/journal.pone.0254910 (PMC8880863; doi:10.1371/journal.pone.0254910)
Supplement: S1 Table — Samples listed according to type of experiment, applied technique, and time series of experiment, site, date of collection, number of wood, and abiotic parameters: depth, temperature, salinity, and pH. Abiotic parameters were measured using a Multi 340i sensor WTW. (DOCX) [file pone.0254910.s005.docx]

**S1 Table. Collections and abiotic parameters measured prior collection at wood surface.** Samples listed according to type of experiment, applied technique, time series of experiment, site, date of collection, number of wood, and abiotic parameters: depth, temperature, salinity, and pH. Abiotic parameters were measured using a Multi 340i sensor WTW.

| experiments | technique | site | date | wood # | depth (m) | temperature (°C) | salinity | pH | time series (h) |
| --- | --- | --- | --- | --- | --- | --- | --- | --- | --- |
| host sulfide starvation | microscopy | Sv. Jernej | 03.07.2015 | 86 | 0.3 | 28.3 | 35 | 8.2 | 0 - 72 |
|  |  | Strunjan | 25.07.2015 | 100 | 0.5 | 30.5 | 36 | 8.3 | 0 - 72 |
| host sulfidic condition | microscopy | Strunjan | 20.07.2021 | 121 | 0.5 | 28.4 | 34 | 8.3 | 0 - 108 |
| symbiont sulfide starvation | FISH | Sv. Jernej | 09.-14.7.2014 | 60-68 | 03-1.0 | 23.5-28.2 | 30-31 | 7.8-8.1 | 24 - 72 |
| symbiont sulfide starvation | SEM micrographs/analyses | Sv. Jernej | 03.10.2012 | 6 | 0.4 | 20.0-20.7 | 34 | 8.1 | 0 (in situ) |
|  | SEM micrographs/analyses |  | 21.10.2012 | 25, 28, 29 | 0.4 | 20.0-20.7 | 34 | 8.1 | 0 - 48 |
|  | SEM micrographs |  | 07.07.2013 | 41 | 0.5 | 28.1 | 34 | 8.1 | 48 |
