# Supplementary material for: Host-symbiont stress response to lack-of-sulfide in the giant ciliate mutualism
Source: PLoS One. 2022 Feb 25;17(2):e0254910. doi: 10.1371/journal.pone.0254910 (PMC8880863; doi:10.1371/journal.pone.0254910)
Supplement: S2 Table — Abiotic parameters: temperature, salinity, pH, and oxygen and sulfide concentrations (mean ± standard deviation). Temperature, salinity, pH were measured using a Multi 340i sensor WTW. Oxygen concentration was measured using a PreSenS Flow-through Cell FTC-PSt3. Sulfide concentration was measured photometrically according to Cline (1969). (DOCX) [file pone.0254910.s006.docx]

**S2 Table. Abiotic parameters measured at the start (and at the end) of experiments.** Abiotic parameters: temperature, salinity, pH and oxygen and sulfide concentrations (mean ± standard deviation). Temperature, salinity, pH were measured using a Multi 340i sensor WTW. Oxygen concentration was measured using a PreSenS Flow-through Cell FTC-PSt3. Sulfide concentration was measured photometrically according to Cline (1969).

| experiments | water | temperature (°C) | salinity | pH | oxygen (% saturation) | ΣH_2_S (µM) |
| --- | --- | --- | --- | --- | --- | --- |
| host sulfide starvation | new | 26.0 ± 0.9 | 36 ± 0 | 8.0 ± 0.1 | 97 ± 6 | N/A |
|  | removed | 25.5 ± 0.6 | 36 ± 1 | 7.7 ± 0.2 | 99 ± 4 | N/A |
| host sulfidic condition | new | 26.8 ± 0.5 | 35 ± 0 | 7.4 ± 0.1 | 4 ± 4 | 448 ± 11 |
|  | removed | 25.4 ± 0.6 | 36 ± 1 | 7.6 ± 0.4 | 89 ± 10 | 6 ± 5 |
| symbiont sulfide starvation | FISH start | 24.2 ± 0.5 | 32 ± 0 | 8.1 ± 0.0 | 101 ± 2 | N/A |
|  | SEM & analyses start | 22.1 ± 0.1 | 34 ± 0 | 8.1 ± 0.0 | 123 ± 0 | N/A |
|  | SEM start | 25.3 ± 1.0 | 36 ± 0 | 8.1 ± 0.0 | 101 ± 3 | N/A |
