# Supplementary material for: Host-symbiont stress response to lack-of-sulfide in the giant ciliate mutualism
Source: PLoS One. 2022 Feb 25;17(2):e0254910. doi: 10.1371/journal.pone.0254910 (PMC8880863; doi:10.1371/journal.pone.0254910)
Supplement: Attachment — Submitted filename: Response to Reviewers.docx [file pone.0254910.s008.docx]

Dear Editor,	Vienna, December 9, 2021

We are happy that the reviewer is satisfied at large with our corrections. Now we just clarified one descriptive paragraph and slightly changed figure 1. We hope now that the manuscript can be accepted for publication.

Yours sincerely

Salvador Manuel Espada Hinojosa on behalf of all the authors

---------------

> Journal Requirements:

>

> Please review your reference list to ensure that it is complete and

> correct. If you have cited papers that have been retracted, please

> include the rationale for doing so in the manuscript text, or remove

> these references and replace them with relevant current references. Any

> changes to the reference list should be mentioned in the rebuttal letter

> that accompanies your revised manuscript. If you need to cite a

> retracted article, indicate the article's retracted status in the

> References list and also include a citation and full reference for the

> retraction notice.

>

> [Note: HTML markup is below. Please do not edit.]

>

> Reviewers' comments:

>

> Reviewer's Responses to Questions

>

> Comments to the Author

>

> 1. If the authors have adequately addressed your comments raised in a

> previous round of review and you feel that this manuscript is now

> acceptable for publication, you may indicate that here to bypass the

> "Comments to the Author" section, enter your conflict of interest

> statement in the "Confidential to Editor" section, and submit your

> "Accept" recommendation.

>

> Reviewer #1: All comments have been addressed

> -------------------------

>

> 2. Is the manuscript technically sound, and do the data support the

> conclusions?

>

> The manuscript must describe a technically sound piece of scientific

> research with data that supports the conclusions. Experiments must have

> been conducted rigorously, with appropriate controls, replication, and

> sample sizes. The conclusions must be drawn appropriately based on the

> data presented.

>

> Reviewer #1: (No Response)

> -------------------------

>

> 3. Has the statistical analysis been performed appropriately and

> rigorously?

>

> Reviewer #1: Yes

> -------------------------

>

> 4. Have the authors made all data underlying the findings in their

> manuscript fully available?

>

> The PLOS Data policy [2] requires authors to make all data underlying

> the findings described in their manuscript fully available without

> restriction, with rare exception (please refer to the Data Availability

> Statement in the manuscript PDF file). The data should be provided as

> part of the manuscript or its supporting information, or deposited to a

> public repository. For example, in addition to summary statistics, the

> data points behind means, medians and variance measures should be

> available. If there are restrictions on publicly sharing data--e.g.

> participant privacy or use of data from a third party--those must be

> specified.

>

> Reviewer #1: Yes

> -------------------------

>

> 5. Is the manuscript presented in an intelligible fashion and written in

> standard English?

>

> PLOS ONE does not copyedit accepted manuscripts, so the language in

> submitted articles must be clear, correct, and unambiguous. Any

> typographical or grammatical errors should be corrected at revision, so

> please note any specific errors here.

>

> Reviewer #1: Yes

> -------------------------

>

> 6. Review Comments to the Author

>

> Please use the space provided to explain your answers to the questions

> above. You may also include additional comments for the author,

> including concerns about dual publication, research ethics, or

> publication ethics. (Please upload your review as an attachment if it

> exceeds 20,000 characters)

>

> Reviewer #1: It's a good revision. I recommend acceptance. However, I

> ask the authors to consider the following suggestions.

>

> line 81. Addition of Fig. 1 has helped me to understand what the

> symbiosis looks like, but for readers who are unfamiliar with it (such

> as me) zooids are still not well described upfront. My dictionary

> defines zooids as individual units of colony, but in the present case

> the description could be more precise. That would make the paper more

> accessible. Please consider something like: "Each colony consists of a

> central stalk of the giant ciliate Zoothamnium niveum and side branches

> that are additional cells of Z. niveum. Attached to the branches are

> zooids that consist each of a central cell of Z. niveum covered with

> ectosymbiotic bacteria. Zooids are of two types. Microzoids occur along

> the branches are vegetative, providing nutrients and energy to the other

> colony parts. Macrozooids develop at the base of each branch and are

> released as "swarmers" for reproductive and dispersal functions."

> The above description may not be accurate, but something similar would

> be helpful to readers upon their first reading of the paper. By the time

> the reader sees Fig. 4 it becomes clearer, I suppose.

> S Tables, how did you measure sulfide and salinity? Probably you can

> insert that information as a footnote to a Table.

**We tried to clarify and changed this paragraph to:**

**L 81 Zoothamnium colonies consist of a stalk and alternating branches on which individual cells grow: feeding cells called microzooids, dividing cells called terminal zooids, and macrozooids, cells responsible for asexual reproduction ([11], Fig 1). The vertical transmission of the ectosymbiont is through macrozooids. These host propagules are released as swarmers into the pelagial for dispersal. Once settled, the swarmer transforms into the terminal zooid and begins to produce the stalk and to divide, producing the terminal zooid of each branch. Nourishing microzooids are produced through division of the terminal zooid on each branch, increasing the length of the branch. Macrozooids develop at the base of the branch. These macrozooids leave the colony as swarmers as soon as a ciliary band has formed.**

> Fig. 1, in the figure the base of the stalk is black on black and does

> not show. Was there a last minute color reversal? (I.e.- a negative

> image.) To me, S1Fig is more helpful.

**In figure 1 we outlined the lower part of the stalk now, which is barely visible due to the lack of the white symbionts.**

**We added in the description:**

**L98 Note that the lower part of the stalk, lacking white symbionts, is outlined.**

> S Tables, how did you measure sulfide and salinity? Probably you can

> insert that information as a footnote to a Table.

We described in more detail how we measured sulfide and salinity in Material and Methods already but added also now in the Supplementary Table descriptions the following:

S1 Table. Collections and abiotic parameters measured prior collection at wood surface. Samples listed according to type of experiment, applied technique, and time series of experiment, site, date of collection, number of wood, and abiotic parameters: depth, temperature, salinity, and pH. Abiotic parameters were measured using a Multi 340i sensor WTW.

 S2 Table. Abiotic parameters measured at the start (and at the end) of experiments. Abiotic parameters: temperature, salinity, pH, and oxygen and sulfide concentrations (mean ± standard deviation). Temperature, salinity, pH were measured using a Multi 340i sensor WTW. Oxygen concentration was measured using a PreSenS Flow-through Cell FTC-PSt3. Sulfide concentration was measured photometrically according to Cline (1969).

(DOCX)

>

> 7. PLOS authors have the option to publish the peer review history of

> their article (what does this mean? [3]). If published, this will

> include your full peer review and any attached files.

>

> If you choose "no", your identity will remain anonymous but your review

> may still be made public.

>

> Do you want your identity to be public for this peer review? For

> information about this choice, including consent withdrawal, please see

> our Privacy Policy [4].

>

> Reviewer #1: No

>

> [NOTE: If reviewer comments were submitted as an attachment file, they

> will be attached to this email and accessible via the submission site.

> Please log into your account, locate the manuscript record, and check

> for the action link "View Attachments". If this link does not appear,

> there are no attachment files.]

>

> While revising your submission, please upload your figure files to the

> Preflight Analysis and Conversion Engine (PACE) digital diagnostic tool,

> https://pacev2.apexcovantage.com/. PACE helps ensure that figures meet

> PLOS requirements. To use PACE, you must first register as a user.

> Registration is free. Then, login and navigate to the UPLOAD tab, where

> you will find detailed instructions on how to use the tool. If you

> encounter any issues or have any questions when using PACE, please email

> PLOS at figures@plos.org. Please note that Supporting Information files

> do not need this step.
